# Myocardial T2* Imaging at 3T and 1.5T: A Pilot Study with Phantom and Normal Myocardium

**DOI:** 10.3390/jcdd9080271

**Published:** 2022-08-16

**Authors:** Suyon Chang, Jinho Park, Young-Joong Yang, Kyongmin Sarah Beck, Pan Ki Kim, Byoung Wook Choi, Jung Im Jung

**Affiliations:** 1 Department of Radiology, Seoul St. Mary’s Hospital, College of Medicine, The Catholic University of Korea, Seoul 06591, Korea; 2 Phantomics, Inc., Seoul 07803, Korea; 3 Department of Radiology, Center for Clinical Imaging Data Science, Research Institute of Radiological Sciences, Severance Hospital, Yonsei University College of Medicine, Seoul 03722, Korea

**Keywords:** magnetic resonance imaging, heart, iron overload, 3T, T2*

## Abstract

Background: Myocardial T2* mapping at 1.5T remains the gold standard, but the use of 3T scanners is increasing. We aimed to determine the conversion equations in different scanners with clinically available, vendor-provided T2* mapping sequences using a phantom and evaluated the feasibility of the phantom-based conversion method. Methods: T2* of a phantom with FeCl_3_ (five samples, 3.53–20.09 mM) were measured with 1.5T (MR-A1) and 3T scanners (MR-A2, A3, B), and the site-specific equation was determined. T2* was measured in the interventricular septum of three healthy volunteers at 1.5T (T2*_1.5T_, MR-A1) and 3T (T2*_3.0T_, MR-B). T2*_3.0T_ was converted based on the equation derived from the phantom (T2*_eq_). Results: R2* at 1.5T and 3T showed linear association, but a different relationship was observed according to the scanners (MR-A2, R2*_1.5T_ = 0.76 × R2*_3.0T_ − 2.23, *R*^2^ = 0.999; MR-A3, R2*_1.5T_ = 0.95 × R2*3.0T − 34.28, *R*^2^ = 0.973; MR-B, R2*_1.5T_ = 0.76 × R2*_3.0T_ − 3.02, *R*^2^ = 0.999). In the normal myocardium, T2*_eq_ and T2*_1.5T_ showed no significant difference (35.5 ± 3.5 vs. 34.5 ± 1.2, *p* = 0.340). The mean squared error between T2*_eq_ and T2*_1.5T_ was 16.33, and Bland–Altman plots revealed a small bias (−0.94, 95% limits of agreement: −8.86–6.99). Conclusions: a phantom-based, site-specific equation can be utilized to estimate T2* values at 1.5T in centers where only 3T scanners are available.

## 1. Introduction

Myocardial T2* mapping is a noninvasive and robust method that is used to identify myocardial iron accumulation in iron storage diseases [1,2]. It is reproducible over time; therefore, it can also be used to monitor chelation therapy [3]. Myocardial T2* mapping has been widely used and validated at 1.5T [4], and currently, 1.5T remains the gold standard for clinical practice [5].

Recently, the use of 3T scanners has been increasing, given their advantages of higher signal-to-noise ratio and scan time, and the number of institutions with only 3T scanners is increasing. Considering this, it is important to accurately calculate the iron burden at 3T. Quantification of myocardial T2* at 3T is challenging because of substantially decreased T2* values and vulnerable quantitation with higher susceptibility artifacts [6,7]. Despite these limitations, the feasibility and reproducibility of T2* or its reciprocal R2* (the relaxation rate, R2* (s^−1^) = 1000/T2* (ms)) quantifications at 3T have been previously demonstrated, particularly for interventricular septum [8,9].

R2* is known to be approximately halved from 3T to 1.5T [10]. Several studies were conducted to confirm this relationship in the myocardium but reported different conversion equations for each research [7,8,9,10,11]. Furthermore, each study used a single cardiovascular magnetic resonance (CMR) scanner for each magnetic field strength. Therefore, it is important to determine whether a theoretical conversion using different scanners with clinically available, vendor-provided T2* mapping sequences can be applied in clinical practice.

In addition, a recent study suggested that myocardial T1 values can be standardized among different scanners with a phantom-based correction method [12]. We hypothesized that the relationships of T2* or R2* values between scanners with different field strengths could also be determined using a phantom-based conversion method.

In this pilot study, we aimed (i) to determine the conversion equations in different scanners using a phantom and (ii) to assess the feasibility of the site-specific equation for estimation of T2* values at 1.5T from a 3T scanner in the myocardium.

## 2. Materials and Methods

This experimental study was performed in two tertiary care hospitals (Institutions A and B) from June 2019 to December 2020. It was approved by the institutional review board of both institutions, and informed consent was obtained from the participants.

### 2.1. Image Acquisition for T2* Mapping

**Figure 1:**
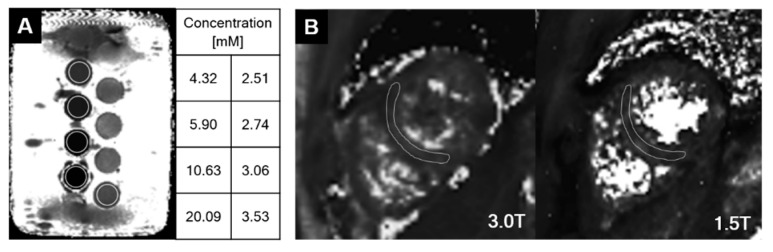
T2* maps in a phantom (**A**) and normal myocardium (**B**).

A phantom with different concentrations of FeCl_3_ was created, and five samples with increasing concentrations of 3.53, 4.32, 5.90, 10.63, and 20.09 mM were used (Figure 1A). The phantom was scanned with four MR scanners (MR-A1, 1.5T, Achieva, Philips Healthcare, Hospital A; MR-A2, 3T, Ingenia CX, Philips Healthcare, Hospital A; MR-A3, 3T, Prisma Fit, Siemens Healthineers, Hospital A; MR-B, 3T, Verio, Siemens Healthineers, Hospital B). In addition, three healthy volunteers with no cardiovascular disease were scanned by MR-A1 (1.5T) and MR-B (3T). T2* maps of the mid-left ventricle were acquired with clinically available, vendor-provided sequences in a short-axis view (Figure 1B). Multi-echo black-blood turbo field echo (Philips) and multi-echo dark blood gradient echo (Siemens) sequences were used. Scan parameters were as follows: in-plane pixel size 1.17–1.34 mm^2^, slice thickness 8–10 mm, repetition time (TR) 14–20 (minimum TR), and number of echoes 6–8. Detailed scan parameters are described in Tables S1 and S2. A small shimming box was used over the heart to correct local magnetic field irregularities.

### 2.2. Image Analysis

The region of interest (ROI) was drawn on T2* maps by two independent cardiothoracic radiologists (S.C. and K.S.B.). Readers were blinded to the results measured by the other reader. All measurements were repeated three times at 1-week intervals. Readers drew circular ROIs as large as possible in the phantom. In human hearts, iron deposition preferentially occurs in the subepicardial area, but no systematic variation occurs between myocardial regions, and the mid-ventricular septal iron is highly representative of global myocardial iron from a previous autopsy study [13]. In addition, in terms of avoiding susceptibility artifacts, ROI in the interventricular septum is recommended for T2* measurement [5]. Therefore, ROIs were drawn at the center of the interventricular septum in this study.

### 2.3. Phantom-Based Equation and Statistical Analysis

R2* has a linear association with the field strength [14]. Therefore, an association between R2* values given by different field strengths was assessed by linear regression with scatter plots for each scanner from the phantom study. Then T2* values at 1.5T can be computed as a function of 3T values from the equation. Intra-class correlation coefficient (ICC) analyses with a 95% confidence interval (CI) were conducted to assess intra- and inter-observer reliability [15]. Intra-observer reliability was assessed using a two-way mixed model with single measures and an absolute agreement. Inter-observer reliability was assessed using a two-way random mixed model with single measures and an absolute agreement. T2* values at 3T (T2*_3.0T_) of healthy volunteers were converted to the equivalent values at 1.5T (T2*_eq_) using the conversion equation obtained from the phantom study using the MR-B scanner. T2*_eq_ and the measured values at 1.5T (T2*_1.5T_) were compared using paired t-test, mean squared error, and Bland–Altman plots. Statistical analyses were performed with SAS version 9.4 (SAS Institute, Cary, CA, USA), SPSS version 24.0 (IBM Corp., Armonk, NY, USA), and R version 4.0.2 (R Core Team, Vienna, Austria).

## 3. Results

### 3.1. Phantom-Based Equation between 1.5T and 3T

Table 1 shows the T2* values of a phantom measured by two independent radiologist readers. T2* values of the phantom ranged from 4.2 to 63.4 msec at 1.5T scanners and from 3.1 to 44.6 msec at 3T scanners. Overall, MR scanners with 3T field strength revealed lower T2* values than those of 1.5T scanners, but values varied according to the MR scanner used.

**Figure 2:**
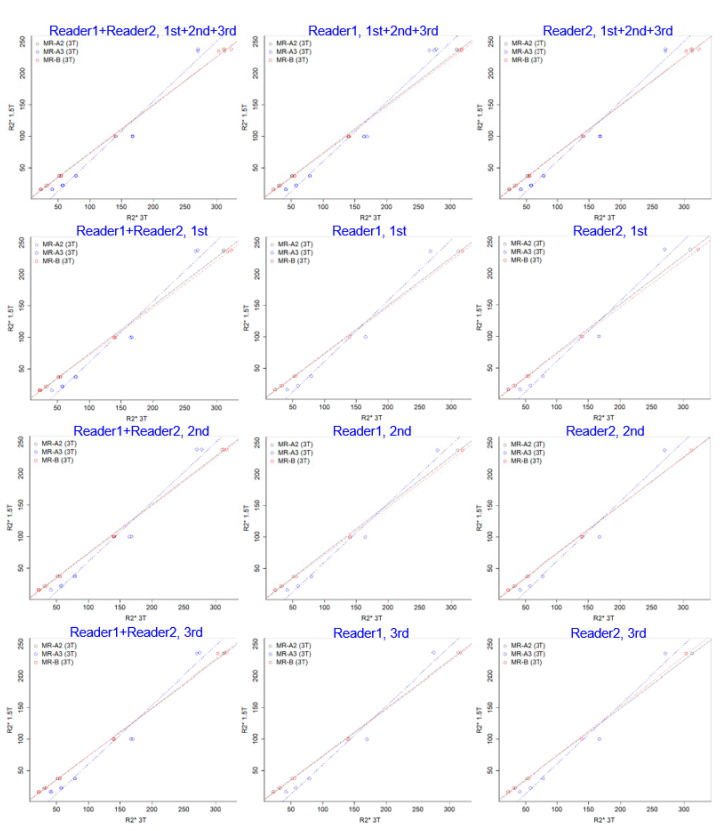
Scatter plots of a phantom with linear regression with repeated measures by two radiologist readers.

The linear association between R2* values at 1.5T and 3T was found with good fit, but a different relationship was observed according to the MR scanners (MR-A2, R2*_1.5T_ = 0.76 × R2*_3.0T_ − 2.23, *R*^2^ = 0.999; MR-A3, R2*_1.5T_ = 0.95 × R2*_3.0T_ − 34.28, *R*^2^ = 0.973; MR-B, R2*_1.5T_ = 0.76 × R2*_3.0T_ − 3.02, *R*^2^ = 0.999; Table 2, Figure 2). 

When comparing both readers, these results were consistently shown in repeated measures. Intra-observer and inter-observer reliability assessment of T2* values demonstrated excellent agreement (ICC range, 0.9997–1.0000 and 0.9993–1.0000, respectively; Table 3).

### 3.2. Normal Myocardium Study

**Figure 3:**
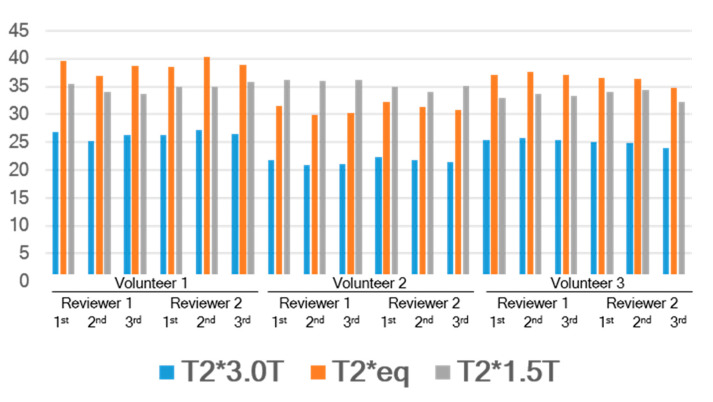
T2* values in three healthy volunteers with repeated measures by two radiologist readers. T2*_3.0T_ = T2* measured at 3T, T2*_eq_ = equivalent T2* calculated from the site-specific equation, T2*_1.5T_= T2* measured at 1.5T.

From the in vivo study with the normal myocardium, no significant susceptibility artifacts were found in the interventricular septum. T2* values ranged from 32.26 to 36.26 at 1.5T scanners and from 20.87 to 27.26 at 3T scanners. The mean and standard deviation of T2*_1.5T_ and T2*_eq_ were 34.5 ± 1.2 (range, 32.3–36.3) and 35.5 ± 3.5 (range, 29.9–40.2), respectively, without any significant statistical difference (*p* = 0.340, Table 4, Figure 3). In addition, there were no significant differences for each reader or each measurement. 

The mean squared error between the predicted T2* values and measured T2* values at 1.5T was 16.33 when considering the two readers. Specifically, this parameter was 21.68 and 10.98 for reader 1 and reader 2, respectively.

**Figure 4:**
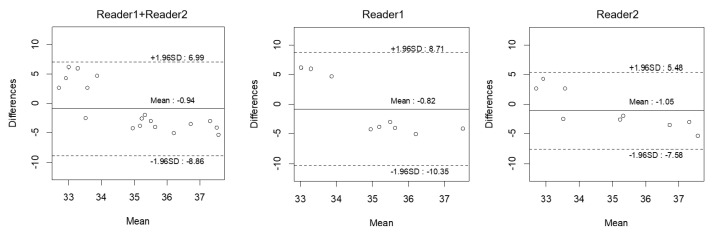
Bland–Altman plots of equivalent T2* values and measured T2* values at 1.5T.

Figure 4 shows the results of Bland–Altman analyses for T2*_1.5T_ and T2*_eq_ for two readers. When considering both readers, Bland–Altman plots revealed a small bias (−0.94, 95% limits of agreement: −8.86, 6.99) between the predicted T2* values and measured T2* values at 1.5T. Moreover, bias remained small when considering each independent reader (bias −0.82, 95% limits of agreement −10.35, 8.71 for reader 1; bias −1.05, 95% limits of agreement −7.58, 5.48 for reader 2).

## 4. Discussion

Our study reveals that different conversion equations are required for different scanners with clinically available, vendor-provided T2* mapping sequences. The phantom-based, site-specific equation enables the estimation of equivalent T2* values at 1.5T from 3T scanners with acceptable mean squared error and small bias, suggesting the feasibility of the phantom-based T2* estimation method in clinical practice. 

T2* mapping has been utilized for iron quantifications in the heart and liver. While calibration of hepatic T2* against liver biopsy has been made for estimation of liver iron concentration [16,17], tissue validation and calibration of myocardial iron measurements remain challenging due to the risks of heart biopsy and inhomogeneous myocardial deposition [18,19]. While one study using a single heart specimen reported a linear association of myocardial R2* and myocardial iron concentration [20], a different study using 12 human heart tissues suggested curvilinear relations between R2* and cardiac iron concentration [13].

Theoretically, R2* has a linear association with the field strength [14]. In the past, there have been multiple attempts to study the relationship of myocardial R2* or T2* at 1.5T and 3T scanners [7,8,9,10,11]. A linear association between R2* and the field strength was suggested in the human myocardium [8,9,10], while others have reported a linear association between the myocardial T2* and the field strength [11]. However, these studies showed different conversion equations or relationships between two field strengths. In addition, a single CMR scanner for each magnetic field strength was used for each study. This suggests that measured values can be different from theoretical calculations in clinical practice. Although it is unclear, one can assume that some acquisition parameters or other scan considerations such as the position in the iso-center or shim volume may affect the measured values. Therefore, it is necessary to evaluate the applicability and feasibility of the theoretical conversion equation in clinically available, vendor-provided T2* mapping sequences. 

In addition, a prior study reported that myocardial T1 values can be standardized among different scanners with a phantom-derived equation [12]. We hypothesized that the relationships of T2* or R2* values between scanners with different field strengths could be determined using a dedicated phantom. We assessed conversion equations using a phantom in different scanners and applied them in the normal myocardium. In our results, the conversion equation differed from the theoretical value (i.e., halving R2* values), while the measured value at 1.5T (T2*_1.5T_) and the calculated value using a site-specific equation (T2*_eq_) in normal myocardium revealed no significant difference. Therefore, we suggest that caution is needed when interpreting T2* values at 3T and that phantom validation is necessary for each scanner in clinical practice.

Our study has some limitations that are important to pinpoint. First, we included only healthy volunteers in the validation experiments. It would have been important to also include patients with iron overload (T2* < 20 msec at 1.5T), given their clinical relevance. Therefore, further investigation of a large number of patients with iron overload is necessary. Second, only a small number of healthy volunteers were included in our study. This has to do with the fact that the normal myocardium study was used only for validation rather than derivation of the equation and that consistent results through the repeated measurements were obtained. Third, the difference between the two high-concentration samples in the phantom (10.63 and 20.09 mM) was high. The relationship could have been derived more accurately with intermediate concentration samples. Fourth, phantom results may not reflect the susceptibility of the tissue. Yet, in our study, there was no evidence of any significant susceptibility artifacts in the interventricular septum. Moreover, the predicted T2* value derived from the site-specific equation did not show a significant difference from the measured value at 1.5T in repeated measures. Hence, we suggest the usage of the site-specific equation only in cases where significant errors are not detected, which can be prevented by proper acquisition and analysis.

## 5. Conclusions

Our study demonstrates that different conversion equations can be required for different scanners with clinically available, vendor-provided T2* mapping sequences. Therefore, caution is needed when interpreting T2* values at 3T, and that phantom validation is necessary for each scanner in clinical practice. A phantom-based, site-specific equation may be utilized to estimate T2* values at 1.5T in centers where only 3T scanners are available. However, further studies involving larger populations and different myocardial iron burdens are needed before its clinical application.

## Figures and Tables

**Table 1:** T2* values in a phantom study measured by two radiologist readers.

Concentration (mM)	Institution	Scanner	Vendor	Field Strength	T2* (Mean ± Standard Deviation)		
					Reader 1 + Reader 2	Reader 1	Reader 2
Overall	A	MR-A1	Philips	1.5T	29.6 ± 22.0	29.5 ± 22.2	29.7 ± 22.5
		MR-A2	Philips	3.0T	21.3 ± 15.7	21.4 ± 16.0	21.3 ± 15.9
		MR-A3	Siemens	3.0T	12.8 ± 7.7	12.8 ± 7.8	12.9 ± 7.9
	B	MR-B	Siemens	3.0T	20.8 ± 15.7	20.8 ± 16.1	20.8 ± 15.9
3.53	A	MR-A1	Philips	1.5T	62.5 ± 0.7	62.0 ± 0.2	62.9 ± 0.7
		MR-A2	Philips	3.0T	44.2 ± 0.3	44.4 ± 0.3	44.0 ± 0.3
		MR-A3	Siemens	3.0T	24.4 ± 0.2	24.3 ± 0.3	24.5 ± 0.2
	B	MR-B	Siemens	3.0T	45.5 ± 0.4	45.8 ± 0.1	45.2 ± 0.2
4.32	A	MR-A1	Philips	1.5T	44.5 ± 0.1	44.5 ± 0.1	44.5 ± 0.0
		MR-A2	Philips	3.0T	32.6 ± 0.2	32.5 ± 0.2	32.8 ± 0.1
		MR-A3	Siemens	3.0T	17.4 ± 0.2	17.3 ± 0.1	17.5 ± 0.2
	B	MR-B	Siemens	3.0T	29.9 ± 0.1	29.9 ± 0.1	29.9 ± 0.1
5.90	A	MR-A1	Philips	1.5T	26.8 ± 0.1	26.8 ± 0.2	26.8 ± 0.2
		MR-A2	Philips	3.0T	19.5 ± 0.1	19.5 ± 0.1	19.5 ± 0.1
		MR-A3	Siemens	3.0T	12.7 ± 0.1	12.7 ± 0.1	12.7 ± 0.1
	B	MR-B	Siemens	3.0T	18.2 ± 0.1	18.2 ± 0.1	18.3 ± 0.1
10.63	A	MR-A1	Philips	1.5T	10.0 ± 0.0	10.0 ± 0.0	10.0 ± 0.0
		MR-A2	Philips	3.0T	7.2 ± 0.1	7.2 ± 0.1	7.1 ± 0.1
		MR-A3	Siemens	3.0T	6.0 ± 0.1	6.0 ± 0.1	6.0 ± 0.0
	B	MR-B	Siemens	3.0T	7.2 ± 0.0	7.2 ± 0.1	7.2 ± 0.0
20.09	A	MR-A1	Philips	1.5T	4.2 ± 0.0	4.2 ± 0.0	4.2 ± 0.1
		MR-A2	Philips	3.0T	3.2 ± 0.0	3.2 ± 0.0	3.2 ± 0.0
		MR-A3	Siemens	3.0T	3.7 ± 0.1	3.7 ± 0.1	3.7 ± 0.0
	B	MR-B	Siemens	3.0T	3.2 ± 0.1	3.2 ± 0.0	3.2 ± 0.1

**Table 2:** Linear regression model of 1.5T on 3.0T.

Institution	Scanner	Vendor	Field Strength	Reader 1 + Reader 2		Reader 1		Reader 2	
				Model	R-Square	Model	R-Square	Model	R-Square
1st + 2nd + 3rd									
A	MR-A2	Philips	3.0T	−2.23 + 0.76 × 3.0T	0.999	−2.23 + 0.76 × 3.0T	0.999	−2.23 + 0.76 × 3.0T	0.999
	MR-A3	Siemens	3.0T	−34.28 + 0.95 × 3.0T	0.973	−34.23 + 0.95 × 3.0T	0.975	−34.33 + 0.95 × 3.0T	0.972
B	MR-B	Siemens	3.0T	−3.02 + 0.76 × 3.0T	0.999	−2.75 + 0.75 × 3.0T	1	−3.30 + 0.76 × 3.0T	0.999
1st									
A	MR-A2	Philips	3.0T	−2.45 + 0.77 × 3.0T	0.999	−2.18 + 0.76 × 3.0T	0.999	−2.73 + 0.77 × 3.0T	0.999
	MR-A3	Siemens	3.0T	−35.28 + 0.96 × 3.0T	0.972	−35.59 + 0.97 × 3.0T	0.973	−34.99 + 0.96 × 3.0T	0.972
B	MR-B	Siemens	3.0T	−2.37 + 0.75 × 3.0T	1	−2.52 + 0.75 × 3.0T	1	−2.23 + 0.74 × 3.0T	1
2nd									
A	MR-A2	Philips	3.0T	−2.42 + 0.77 × 3.0T	0.999	−2.60 + 0.77 × 3.0T	0.999	−2.24 + 0.76 × 3.0T	0.999
	MR-A3	Siemens	3.0T	−33.89 + 0.95 × 3.0T	0.975	−33.66 + 0.94 × 3.0T	0.98	−34.17 + 0.95 × 3.0T	0.971
B	MR-B	Siemens	3.0T	−3.28 + 0.76 × 3.0T	0.999	−3.16 + 0.76 × 3.0T	0.999	−3.42 + 0.77 × 3.0T	0.999
3rd									
A	MR-A2	Philips	3.0T	−1.84 + 0.75 × 3.0T	0.999	−1.94 + 0.75 × 3.0T	0.999	−1.73 + 0.75 × 3.0T	1
	MR-A3	Siemens	3.0T	−33.72 + 0.94 × 3.0T	0.973	−33.65 + 0.93 × 3.0T	0.973	−33.84 + 0.95 × 3.0T	0.974
B	MR-B	Siemens	3.0T	−3.47 + 0.77 × 3.0T	0.999	−2.58 + 0.75 × 3.0T	1	−4.54 + 0.79 × 3.0T	0.999

**Table 3:** Results of intra-observer and inter-observer reproducibility analyses for the phantom study.

Scanner	Intra-Observer Reliability		Inter-Observer Reliability			
	Reader 1	Reader 2	1st + 2nd + 3rd	1st	2nd	3rd
MR-A1	1	0.9998	0.9997	0.9996	1	0.9996
	(0.9998–1.0000)	(0.9991–1.0000)	(0.9991–0.9999)	(0.9971–1.0000)	(0.9998–1.0000)	(0.9971–1.0000)
MR-A2	0.9999	0.9999	0.9998	0.9997	0.9999	0.9999
	(0.9994–1.0000)	(0.9997–1.0000)	(0.9995–0.9999)	(0.9978–1.0000)	(0.9995–1.0000)	(0.9992–1.0000)
MR-A3	0.9997	0.9999	0.9995	0.9999	0.9996	0.9993
	(0.9986–1.0000)	(0.9994–1.0000)	(0.9986–0.9998)	(0.9993–1.0000)	(0.9967–1.0000)	(0.9946–0.9999)
MR-B	1	1	0.9998	0.9999	0.9998	0.9999
	(1.0000–1.0000)	(0.9998–1.0000)	(0.9995–0.9999)	(0.9995–1.0000)	(0.9983–1.0000)	(0.9991–1.0000)

Data are intra-class correlation coefficient (95% confidence interval).

**Table 4:** Comparison of T2* measured in 1.5T (T2*_1.5T_) and equivalent T2* values from 3.0T (T2*_eq_) in normal myocardium.

Values	Reader 1 + Reader 2			Reader 1			Reader 2		
	T2*	Difference	*p*-Value	T2*	Difference	*p*-Value	T2*	Difference	*p*-Value
	(Mean ± SD)	(95% CI)		(Mean ± SD)	(95% CI)		(Mean ± SD)	(95% CI)	
1st + 2nd + 3rd									
T2*_1.5T_	34.5 ± 1.2	−0.94	0.34	34.6 ± 1.4	−0.82	0.625	34.5 ± 1.0	−1.05	0.373
T2*_eq_	35.5 ± 3.5	(−2.95–1.08)		35.4 ± 3.8	(−4.56–2.91)		35.5 ± 3.4	(−3.61–1.51)	
1st									
T2 *_1.5T_	34.7 ± 1.2	−1.19	0.484	34.8 ± 1.8	−1.22	0.721	34.6 ± 0.6	−1.16	0.605
T2*_eq_	35.9 ± 3.3	(−5.23–2.85)		36.0 ± 4.1	(−13.9–11.50)		35.8 ± 3.2	(−9.34–7.03)	
2nd									
T2*_1.5T_	34.5 ± 0.9	−0.93	0.626	34.6 ± 1.3	−0.29	0.937	34.4 ± 0.4	−1.58	0.566
T2*_eq_	35.4 ± 3.9	(−5.55–3.69)		34.9 ± 4.3	(−14.2–13.58)		36.0 ± 4.4	(−11.5–8.38)	
3rd									
T2*_1.5T_	34.4 ± 1.6	−0.69	0.73	34.4 ± 1.6	−0.96	0.809	34.4 ± 1.9	−0.42	0.876
T2*_eq_	35.1 ± 3.8	(−5.53–4.15)		35.4 ± 4.5	(−16.0–14.04)		34.8 ± 4.0	(−10.522129.67)	

CI: confidence interval; SD: standard deviation.

## Data Availability

The data presented in this study are available on request from the corresponding author.

## Supplementary Materials

The following supporting information can be downloaded at: https://www.mdpi.com/article/10.3390/jcdd9080271/s1, Table S1: T2* mapping scan parameters; Table S2: Repetition time (TR) and echo time (TE).
