# Supplementary material for: Myocardial T2* Imaging at 3T and 1.5T: A Pilot Study with Phantom and Normal Myocardium
Source: J Cardiovasc Dev Dis. 2022 Aug 16;9(8):271. doi: 10.3390/jcdd9080271 (PMC9410052; doi:10.3390/jcdd9080271)
Supplement: Supplementary file 1 [file jcdd-09-00271-s001.zip › jcdd-1839203-supplementary.pdf]

Journal of
Cardiovascular Development and Disease

Supplementary Tables

Table S1. T2* mapping scan parameters

Scanner	Institution	Field strength	Manufacturer	Model name	Flip angle (degree)	FOV	Slice Thickness (mm)	Pixel spacing (mm^2)	Matrix size
MR-A1	A	1.5T	Philips	Achieva dStream	20	300*300	10	1.34*1.34	224*224
MR-A2	A	3.0T	Philips	Ingenia CX	25	300*300	8	1.34*1.34	224*224
MR-A3	A	3.0T	Siemens	Prisma Fit	15	300*196	8	1.17*1.17	256*168
MR-B	B	3.0T	Siemens	Verio	12	380*332	10	1.17*1.17	256*176

FOV: field of view.

Table S2. Repetition time (TR) and echo time (TE)

Scanner	TR (ms)	TE (1)	TE (2)	TE (3)	TE (4)	TE (5)	TE (6)	TE (7)	TE (8)
MR-A1	14.6	1.2	3.2	5.2	7.2	9.3	11.3	13.3	-
MR-A2	14.4	1.1	2.8	4.6	6.3	8	9.7	11.5	13.2
MR-A3	20	1.9	4.4	6.9	9.5	12	14.6	-	-
MR-B	20	2.5	4.8	7.2	9.5	11.8	14.1	16.4	18.8

TE: echo time; TR: repetition time.
